# Comparative effectiveness of omalizumab in asthma-COPD overlap vs. asthma: a retrospective cohort study

**DOI:** 10.3389/fmed.2026.1738610

**Published:** 2026-01-21

**Authors:** Hengxing Sun, Yunlu Gu, Yinghong Wang, Long Chen, Boyu Guo, Xiaolian Song, Feifei Song, Shuanshuan Xie

**Affiliations:** 1 Department of Respiratory and Critical Care Medicine, Shanghai Tenth People’s Hospital, Tongji University School of Medicine, Shanghai, China; 2 Tongji University School of Medicine, Shanghai, China; 3 Department of Pathology, Shanghai Tenth People’s Hospital, Tongji University School of Medicine, Shanghai, China

**Keywords:** asthma, asthma-COPD overlap, omalizumab, serum total IgE, Th2 inflammation

## Abstract

**Background:**

Asthma-chronic obstructive pulmonary disease (COPD) overlap (ACO) represents a heterogeneous phenotype with diagnostic challenges and variable responses to biologic therapies. Omalizumab, an anti-IgE monoclonal antibody, is effective in allergic asthma but shows limited efficacy in ACO, necessitating mechanistic insights into treatment heterogeneity. This study aims to compare the 20-week omalizumab efficacy between ACO and non-ACO asthma patients and to assess how differing ACO diagnostic criteria affect Th2-inflammatory biomarker dynamics and clinical outcomes.

**Methods:**

We retrospectively analyzed the clinical data of asthma patients who received omalizumab therapy at our hospital between March 2024 and January 2025. All enrolled patients had a documented asthma diagnosis according to the Global Initiative for Asthma (GINA) guidelines. Participants were categorized into ACO and non-ACO asthma groups based on two distinct criteria. The ACO-A group was defined by a prior diagnosis or self-reported history of COPD superimposed on asthma. The ACO-B group required a post-bronchodilator (BD) forced expiratory volume in one second to forced vital capacity ratio (post BD FEV_1_/FVC) < 0.7 and a smoking history of ≥10 pack-years in addition to the asthma diagnosis. Serological, airway inflammatory, and pulmonary function biomarkers related to asthma were measured and comparatively analyzed.

**Results:**

A total of 74 patients were enrolled, of whom 25 were ACO-A, 49 were non-ACO-A, 11 were ACO-B, and 63 were non-ACO-B. Patients with ACO exhibited poorer baseline lung function and higher smoking exposure than those with asthma alone. While both groups showed increased asthma control test (ACT) scores, the non-ACO-A group displayed decreased fractional exhaled nitric oxide (FeNO) and eosinophil (EOS) (all *p* < 0.001) and increased serum total IgE, pre-BD FEV_1_%predicted, post-BD FEV_1_%predicted, and post-BD FEV_1_/FVC (all *p* < 0.001). Changes in serum total IgE, FeNO, and pre-BD FEV_1_%predicted (all *p <* 0.05) were greater in the non-ACO-A group than in the ACO-A group.

**Conclusion:**

Our findings demonstrate that the Th2-high inflammatory endotype, rather than the ACO diagnostic label, is the primary predictor of omalizumab response. Prioritizing direct assessment of Th2 inflammation over the ACO definition can better guide biologic therapy.

**Clinical trial registration:**

www.medicalresearch.org.cn/, identifier MR-31-24-055473.

## 1 Introduction

Asthma-chronic obstructive pulmonary disease (COPD) overlap syndrome (ACOS) is characterized by the presence of multiple clinical features of both asthma and COPD (1). This term was formally established in the 2015 joint report by the Global Initiative for Asthma (GINA) and the Global Initiative for Chronic Obstructive Lung Disease (GOLD) (2). However, the term “syndrome” implied a single disease entity, which was later recognized as an oversimplification of the complex and heterogeneous nature of these overlapping conditions (3). In 2017, the American Thoracic Society (ATS) and the National Heart, Lung, and Blood Institute (NHLBI) have further refined ACOS as ACO to emphasize that it is not a single disease but a cluster of phenotypes driven by distinct underlying mechanisms (3). These documents highlighted a pragmatic diagnostic framework for ACO, defined by a history of asthma, significant smoking exposure, and persistent airflow obstruction, while acknowledging the need for individualized assessment.

Key clinical manifestations of ACO include persistent airflow obstruction, mixed inflammatory patterns, and concurrent symptoms of both disorders (4). The pathophysiology of ACO involves an interplay between neutrophilic inflammation and Th2-driven eosinophilic inflammation, with elevated Th2 cytokines (e.g., IL-4, IL-5, IL-13) promoting IgE production, eosinophil recruitment, and airway hyperresponsiveness (5–7). This Th2-inflammation contrasts with the neutrophilic inflammation typical of COPD, yet overlaps with the Th2 signature of asthma, contributing to diagnostic and therapeutic complexity.

Although a standardized diagnostic framework for ACO is currently lacking, core diagnostic criteria typically integrate key features of both conditions. The diagnostic approach often differs based on whether the patient is initially assessed from an asthma or a COPD perspective. For patients with a primary diagnosis of COPD, features suggestive of ACO include a documented history of asthma, particularly with onset before age 40, a significant bronchodilator (BD) response evidenced by an increase in forced expiratory volume in one second (FEV_1_) of at least 15%, or the presence of airway hyper-responsiveness. Conversely, in patients with diagnosed asthma, the diagnosis of ACO usually requires a significant smoking history of at least 10 pack-years together with persistent airflow limitation demonstrated by a post-BD FEV_1_ / forced vital capacity (FVC) ratio below 0.7 (8–10).

The reported prevalence of ACO varies widely, likely due to differences in diagnostic criteria (9). Previous studies indicate that the combined prevalence of ACO in the general population, asthma patients, and COPD patients is 2.0, 26.5, and 29.6% respectively (11). Moreover, ACO patients experience a higher disease burden, reduced health-related quality of life, and more frequent and severe exacerbations compared to those with isolated COPD or asthma (8, 12, 13). Management of ACO typically follows a stepwise or escalating regimen based on symptom progression and clinical worsening, similar to asthma or COPD, with pharmacological interventions primarily involving inhaled glucocorticoids (ICS) and bronchodilators (14). Nevertheless, a significant proportion of patients with ACO and severe asthma exhibit poor or absent response to glucocorticoid therapy, a condition often referred to as steroid resistance (15, 16). This resistance considerably limits the efficacy of conventional anti-inflammatory regimens, underscoring the need for alternative therapeutic strategies. In this context, biologics that target specific inflammatory pathways have gained increasing importance as potential treatment options for this challenging patient subgroup (17).

Omallizumab, an anti-IgE monoclonal antibody, has been approved for the treatment of allergic asthma in patients sensitized to perennial allergens and IgE levels between 30 and 700 kU/L (18). Previous studies have shown that omalizumab reduces acute asthma exacerbations, improves Asthma Control Test (ACT) scores, and decreases neutrophil counts (19). A long-term retrospective analysis further indicated that 12 months of omalizumab treatment significantly improved lung function and reduced airway inflammation in patients with severe asthma (20).

However, most omalizumab efficacy studies have focused on populations, with limited investigations in ACO cohorts (21, 22). Emerging evidence suggests that Th2-inflammatory biomarkers may predict the responses to biologics like omalizumab (23, 24), though their utility in ACO remains debated due to confounding neutrophilic inflammation. This retrospective cohort study aimed to evaluate the therapeutic efficacy of omalizumab in non-ACO asthma and ACO patients, while offering new insights into clinical management of ACO.

## 2 Materials and methods

### 2.1 Study design and patients

**Figure 1:**
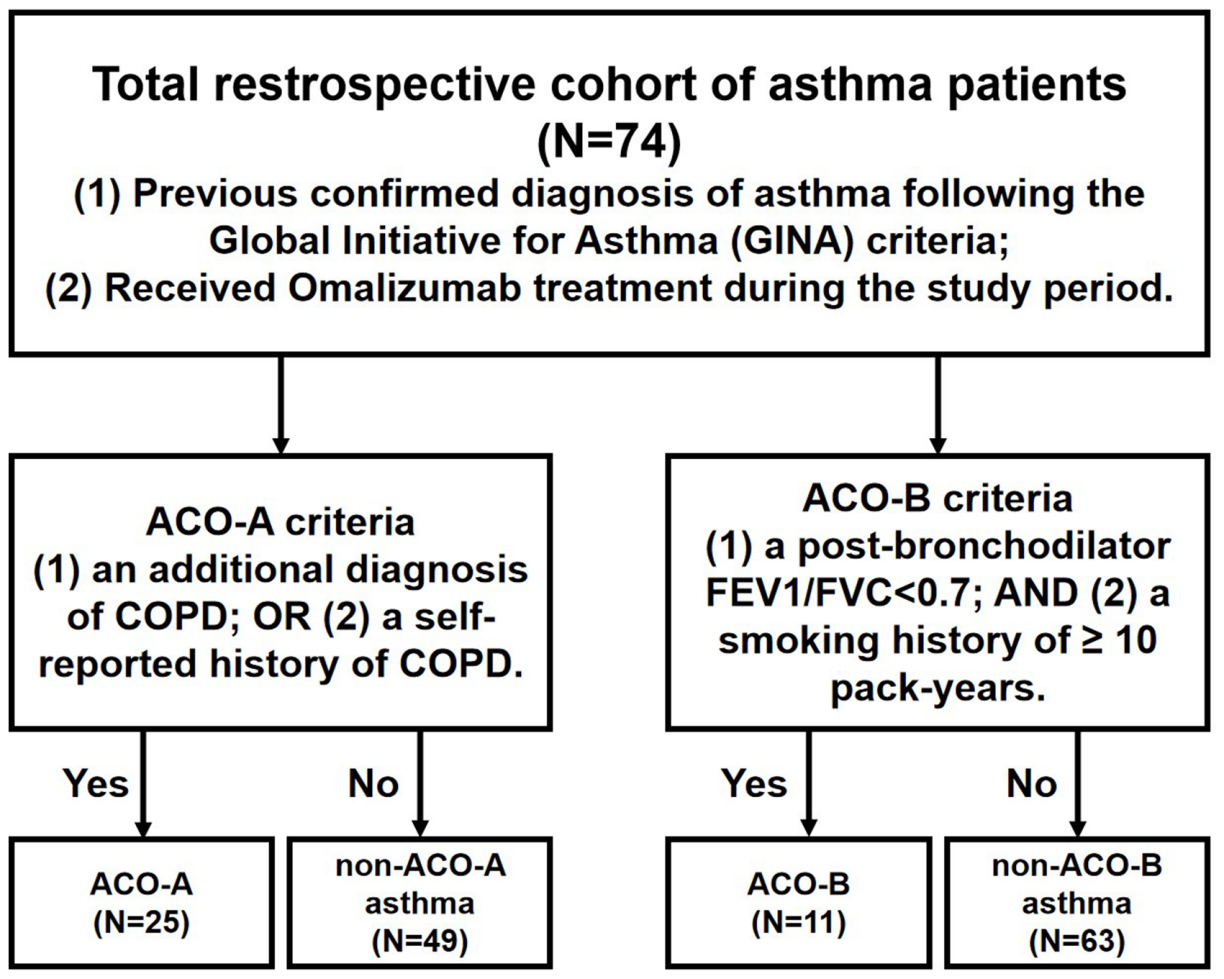
Diagram of research process and patients grouping.

This retrospective cohort study was conducted at Shanghai Tenth People’s Hospital, China. A cohort of patients with asthma was enrolled to investigate the efficacy of omalizumab in ACO and non-ACO asthma subgroups. All participants had a documented diagnosis of asthma for several years. The diagnostic criteria for asthma in this study follow the GINA recommendations. All patients received omalizumab treatment between March 2024 and January 2025 at our institution. Participants with asthma were stratified into two mutually exclusive ACO subgroups based on distinct definitions. ACO-A was defined by: (1) an additional diagnosis of COPD; or (2) a self-reported history of COPD, while ACO-B required both: (1) a post-BD FEV_1_/FVC < 0.7; and (2) a smoking history of ≥ 10 pack-years. Accordingly, the non-ACO-A and non-ACO-B groups comprised asthma patients not meeting the respective ACO criteria (Figure 1). We cautiously noted that there may be heterogeneity in clinical manifestations and pathophysiological mechanisms between non-ACO-A and non-ACO-B group of patients. The purpose of establishing these groups is to compare its clinical characteristics and outcomes with patients who meet the ACO diagnostic criteria. The study was approved by the Ethics Committee of Shanghai Tenth People’s Hospital (No. SHSY-IEC-5.0/24 K196/P01).

### 2.2 Covariates

Baseline demographic and clinical characteristics, including age, sex, age at initial asthma diagnosis, body mass index (BMI), smoking history, current therapy, comorbidities, number of acute exacerbations during one month, ACT scores, eosinophil (EOS) count, serum total IgE, fractional exhaled nitric oxide (FeNO), and lung function test indicators, were collected and analyzed. In asthma management, EOS counts are key biomarkers for assessing inflammation endotypes and predicting treatment responses to biologics. To evaluate the relationship between Th2 inflammation levels and therapeutic outcomes, patients were stratified into subgroups using thresholds of 150 cells/μL for EOS counts, which are recommended thresholds by GINA for identifying Th2 inflammation in asthma. “Acute exacerbation” is defined as an acute worsening of patients’ baseline respiratory symptoms (such as dyspnea, cough, or sputum production) that necessitates additional medical treatment. Lung function parameters included pre-bronchodilator forced expiratory volume in one second (pre-BD FEV_1_%predicted), post-bronchodilator forced expiratory volume in one second (post-BD FEV_1_%predicted), the post-bronchodilator forced expiratory volume in one second to forced vital capacity ratio (post-BD FEV_1_/FVC), and BD reversibility.

### 2.3 Statistical analysis

Numerical variables are presented as mean with standard deviation (SD) for approximately normally distributed data, or as median with interquartile range (IQR) for non-normally distributed data. Comparison of numerical variables before and after treatment within each subgroup was conducted using paired t test (the difference conforms to a normal distribution) or the Wilcoxon signed rank test (the difference conforms to a non-normal distribution). Wilcoxon test and Student’s *t*-test were used for group comparisons. Categorical variables were presented as frequencies and percentages, and the chi-square test and Fisher’s exact test were used for comparisons between the two groups. An analysis of covariance (ANCOVA) was performed to compare outcomes for ACO-A versus non-ACO-A, adjusting for baseline age, which differed significantly between the groups. All analyses were performed using the R software (version 4.4.1), SPSS (version 26.0) and GraphPad Prism (version 9.5.0). All significance tests were two-tailed, and *p* values < 0.05 were considered statistically significant.

## 3 Results

### 3.1 Baseline participant characteristics

A total of 74 asthma patients were recruited in this study, including 25 in the ACO-A group and 49 in the non-ACO-A group, as well as 11 in the ACO-B group and 63 in the non-ACO-B group. The patient evaluation and grouping scheme are summarized in Figure 1. Compared to the non-ACO-A group, the ACO-A group was significantly older [69.00 (63.00–73.00) vs. 55.00 (38.00–66.00), *p* < 0.001], and had lower pre-BD FEV_1_%predicted (44.04 ± 14.14 vs. 77.71 ± 18.43, *p* < 0.001), lower post-BD FEV_1_%predicted (49.56 ± 15.36 vs. 84.03 ± 18.68, *p* < 0.001) and lower post-BD FEV_1_/FVC [68.80 (61.00–72.30) vs. 98.60 (86.90–103.70), *p* < 0.001]. Similarly, the ACO-B group showed significantly lower pre-BD FEV_1_%predicted (42.95 ± 16.65 vs. 70.42 ± 22.01, *p* < 0.001), lower post-BD FEV_1_%predicted (47.96 ± 17.17 vs. 76.65 ± 22.52, *p* < 0.001) and lower post-BD FEV_1_/FVC [61.10 (53.80–70.05) vs. 93.20 (84.30–103.40), *p* < 0.001] compared to patients in non-ACO-B group. Overall, the ACO groups included significantly more smokers than the non-ACO groups (*p* < 0.001). Current therapy also differed significantly between ACO and non-ACO groups. Additional details are listed in Table 1.

**Table 1:** Baseline characteristics in ACO and non-ACO asthma patients with omalizumab treatment.

Variables	Total (*n* = 74)	ACO-A (*n* = 25)	non-ACO-A (*n* = 49)	*P*	ACO-B (*n* = 11)	non-ACO-B (*n* = 63)	*P*
Sex				0.215			0.202
Female	40 (54.05)	11 (44.00)	29 (59.18)		4 (36.36)	36 (57.14)	
Male	34 (45.95)	14 (56.00)	20 (40.82)		7 (63.64)	27 (42.86)	
Age (years)	63.00 (42.50–69.75)	69.00 (63.00–73.00)	55.00 (38.00–66.00)	< 0.001	69.00 (65.00–70.50)	62.00 (41.00–68.00)	0.068
Age at diagnosis (years)	49.00 (36.25–63.75)	54.00 (38.00–65.00)	45.00 (36.00–63.00)	0.407	59.00 (41.50–64.00)	48.00 (36.00–63.50)	0.616
BMI (kg/m^2^)	23.90 (22.00–26.60)	23.70 (21.50–25.20)	24.20 (22.40–26.90)	0.258	24.10 (19.90–25.95)	23.90 (22.20–26.55)	0.438
Smoking				^#^< 0.001			^#^< 0.001
Never	55 (74.32)	12 (48.00)	43 (87.76)		0 (0.00)	55 (87.30)	
Former	9 (12.16)	5 (20.00)	4 (8.16)		4 (36.36)	5 (7.94)	
Current	10 (13.51)	8 (32.00)	2 (4.08)		7 (63.64)	3 (4.76)	
Current therapy				0.010			0.004
ICS-LABA	38 (51.35)	8 (32.00)	30 (61.22)		3 (27.27)	35 (55.56)	
IVGC	22 (29.73)	13 (52.00)	9 (18.37)		8 (72.72)	14 (22.22)	
Neither	14 (18.92)	4 (16.00)	10 (20.41)		0 (0.00)	14 (22.22)	
Comorbidities							
Hypertension	22 (29.73)	11 (44.00)	11 (22.45)	0.055	4 (36.36)	18 (28.57)	0.870
Diabetes mellitus	10 (13.51)	7 (28.00)	3 (6.12)	0.025	2 (18.18)	8 (12.70)	0.990
Tumor	4 (5.41)	3 (12.00)	1 (2.04)	0.212	2 (18.18)	2 (3.17)	0.103
Coronary heart disease	8 (10.81)	3 (12.00)	5 (10.20)	1.00	1 (9.09)	7 (11.11)	1.000
Allergic rhinitis	11 (14.86)	2 (8.00)	9 (18.37)	0.314	2 (18.18)	9 (14.29)	1.000
Acute exacerbations during 1 month (times)				^#^0.215			^#^1.000
0	6 (8.11)	2 (8.00)	4 (8.16)		1 (9.09)	5 (7.94)	
1–3	60 (81.08)	18 (72.00)	42 (85.71)		9 (81.82)	51 (80.95)	
≥4	8 (10.81)	5 (20.00)	3 (6.12)		1 (9.09)	7 (11.11)	
ACT scores	20.00 (17.00–22.75)	20.00 (16.00–23.00)	20.00 (18.00–22.00)	0.605	20.00 (17.00–21.00)	20.00 (17.00–23.00)	0.302
Laboratory findings							
EOS (/uL)	240.00 (120.00–440.00)	170.00 (100.00–420.00)	250.00 (140.00–440.00)	0.394	130.00 (60.00–43.00)	250.00 (140.00–430.00)	0.189
EOS group (/uL)				0.129			^#^0.041
<150	24 (32.43)	11 (44.00)	13 (26.53)		7 (63.64)	17 (26.98)	
≥150	50 (67.57)	14 (56.00)	36 (73.47)		4 (36.36)	46 (73.02)	
Serum total IgE (UI/mL)	434.00 (238.75–1027.50)	437.00 (242.00–1050.00)	431.00 (227.00–1020.00)	0.806	608.00 (270.50–718.50)	431.00 (232.50–1053.45)	0.879
FeNO (ppb)	30.00 (21.25–62.50)	29.00 (22.00–36.00)	30.00 (19.00–73.00)	0.511	24.00 (22.00–47.00)	33.00 (20.00–61.00)	0.504
Lung function test							
Pre-BD FEV_1_ (%pred)	66.34 ± 23.37	44.04 ± 14.14	77.71 ± 18.43	< 0.001	42.95 ± 16.65	70.42 ± 22.01	< 0.001
Post-BD FEV_1_ (%pred)	72.39 ± 24.01	49.56 ± 15.36	84.03 ± 18.68	< 0.001	47.96 ± 17.17	76.65 ± 22.52	< 0.001
Post-BD FEV_1_/FVC (%)	88.95 (72.30–103.10)	68.80 (61.00–72.30)	98.60 (86.90–103.70)	< 0.001	61.10 (53.80–70.05)	93.20 (84.30–103.40)	< 0.001
Reversibility (%)	9.29 (2.67–15.46)	11.72 (4.19–23.45)	8.76 (2.22–12.73)	0.244	9.26 (2.52–28.51)	9.32 (3.32–14.82)	0.659

The data is presented in the form of mean ± standard deviation (normal distribution) and median (1st Quartile–3rd Quartile) (non normal distribution). *P* values between two subgroups were derived from the Student’s t test (for normally distributed variables), Mann–Whitney test (for non-normally distributed variables) and chi-square test or fisher exact test (for categorical variables).

^#^For Fisher exact test.

ACO, asthma-chronic obstructive pulmonary disease overlap; ICS, inhaled corticosteroid; LABA, long-acting beta-agonist; IVGC, intravenous glucocorticoid; ACT, asthma control test; EOS, eosinophil; FeNO, fractional exhaled nitric oxide; Pre-BD FEV_1_, pre-bronchodilator forced expiratory volume in 1 s %; Post-BD FEV_1_, post-bronchodilator forced expiratory volume in 1 s %; Post-BD FEV_1_/FVC, post-bronchodilator forced expiratory volume in 1 s/forced vital capacity ratio.

### 3.2 Changes in multiple biomarkers and lung function after 20 weeks of omalizumab treatment in ACO and non-ACO asthma patients

Changes in treatment methods, monthly acute exacerbations, ACT scores, serum total IgE, and EOS count at baseline and after 20 weeks of omalizumab therapy were analyzed in ACO and non-ACO asthma patients, as detailed in Tables 2, 3. The number of patients receiving intravenous corticosteroid therapy significantly decreased in the entire cohort (all *p* < 0.001). The proportion of patients with no acute exacerbations during the first month significantly increased in the ACO-A (*p* < 0.001), non-ACO-A (*p* < 0.001), ACO-B (*p =* 0.002), and non-ACO-B groups (*p* < 0.001). ACT scores also increased significantly in the ACO-A [22.00 (20.00–23.00) vs. 17.00 (16.00–20.00), *p* < 0.001], non-ACO-A [22.00 (20.00–22.00) vs. 19.00 (17.00–21.00), *p* < 0.001], ACO-B [23.00 (19.00–23.50) vs. 18.00 (17.00–20.00), *p* < 0.001], and non-ACO-B groups [22.00 (20.00–22.50) vs. 18.00 (17.00–21.00), *p* < 0.001].

**Table 2:** Comparative effectiveness of omalizumab: Within-group changes from baseline and between-group differences post-treatment in ACO-A and non-ACO-A asthma patients.

Variables	ACOA (*n* = 25)			non-ACOA (*n* = 49)			*P*
	Baseline	Omalizumab	*P*	Baseline	Omalizumab	*P*	
Therapy			< 0.001			^#^< 0.001	^#^0.057
ICS-LABA	8 (32.00)	3 (12.00)		30 (61.22)	9 (18.37)		
IVGC	13 (52.00)	3 (12.00)		9 (18.37)	0 (0.00)		
Neither	4 (16.00)	19 (76.00)		10 (20.41)	40 (81.63)		
Acute exacerbations during 1 month (times)			^#^< 0.001			^#^< 0.001	1.000
0	2 (8.00)	21 (84.00)		4 (8.16)	42 (85.71)		
1–3	18 (72.00)	4 (16.00)		42 (85.71)	7 (14.29)		
≥4	5 (20.00)	0 (0.00)		3 (6.12)	0 (0.00)		
ACT scores	17.00 (16.00–20.00)	22.00 (20.00–23.00)	< 0.001	19.00 (17.00–21.00)	22.00 (20.00–22.00)	< 0.001	0.822
Laboratory findings							
EOS (/uL)	170.00 (100.00–420.00)	200.00 (80.00–280.00)	0.541	250.00 (140.00–440.00)	120.00 (60.00–210.00)	< 0.001	0.274
EOS group (/uL)			0.777			< 0.001	0.129
<150	11 (44.00)	12 (48.00)		13 (26.53)	31 (63.27)		
≥150	14 (56.00)	13 (52.00)		36 (73.47)	18 (36.73)		
Serum total IgE (UI/mL)	437.00 (242.00–1050.00)	469.00 (212.00–1071.00)	0.134	431.00 (227.00–1020.00)	1320.00 (494.00–2380.00)	<0.001	0.068
FeNO (ppb)	29.00 (22.00–36.00)	22.00 (13.50–29.00)	0.044	30.00 (19.00–73.00)	14.00 (8.00–22.00)	< 0.001	0.097
Lung function test							
Pre-BD FEV_1_ (%pred)	44.04 ± 14.14	47.47 ± 11.95	0.035	77.71 ± 18.43	85.89 ± 9.49	< 0.001	< 0.001
Post-BD FEV_1_ (%pred)	49.56 ± 15.36	53.03 ± 12.33	0.062	84.03 ± 18.68	93.72 ± 10.76	< 0.001	< 0.001
Post-BD FEV_1_/FVC (%)	68.80 (61.00–72.30)	67.60 (58.85–75.25)	0.828	98.60 (86.90–103.70)	100.10 (96.80–108.60)	< 0.001	< 0.001
Reversibility (%)	11.72 (4.19–23.45)	13.85 (6.77–20.82)	0.874	8.76 (2.22–12.73)	7.3 (4.4–11.73)	0.429	0.066

The data is presented in the form of mean ± standard deviation (normal distribution) and median (1st Quartile–3rd Quartile) (non normal distribution). *P* values of characteristics before and after omalizumab treatment were derived from the paired *t* test (the difference conforms to a normal distribution), the Wilcoxon signed rank test (the difference conforms to a non-normal distribution) and chi-square test or fisher exact test (for categorical variables). *P* values between two subgroups were derived from the Student’s t test (for normally distributed variables), Mann–Whitney test (for non-normally distributed variables) and chi-square test or fisher exact test (for categorical variables).

^#^For Fisher exact test.

ACO, asthma-chronic obstructive pulmonary disease overlap; ICS, inhaled corticosteroid; LABA, long-acting beta-agonist; IVGC, intravenous glucocorticoid; ACT, asthma control test; EOS, eosinophil; FeNO, fractional exhaled nitric oxide; Pre-BD FEV_1_, pre-bronchodilator forced expiratory volume in 1 s %; Post-BD FEV_1_, post-bronchodilator forced expiratory volume in 1 s %; Post-BD FEV_1_/FVC, post-bronchodilator forced expiratory volume in 1 s/forced vital capacity ratio.

**Table 3:** Comparative effectiveness of omalizumab: Within-group changes from baseline and between-group differences post-treatment in ACO-B and non-ACO-B asthma patients.

Variables	ACO-B (*n* = 11)			non-ACO-B (*n* = 63)			*P*
	Baseline	Omalizumab	*P*	Baseline	Omalizumab	*P*	
Therapy			< 0.001			< 0.001	0.504
ICS-LABA	3 (27.27)	1 (9.09)		35 (55.56)	11 (17.46)		
IVGC	8 (72.73)	1 (9.09)		14 (22.22)	2 (3.17)		
Neither	0 (0.00)	9 (81.82)		14 (22.22)	50 (79.37)		
Acute exacerbations during 1 month (times)			^#^0.002			^#^< 0.001	1.000
0	1 (9.09)	9 (81.82)		5 (7.94)	54 (85.71)		
1–3	9 (81.82)	2 (18.18)		51 (80.95)	9 (14.29)		
≥4	1 (9.09)	0 (0.00)		7 (11.11)	0 (0.00)		
ACT scores	18.00 (17.00–20.00)	23.00 (19.00–23.50)	< 0.001	18.00 (17.00–21.00)	22.00 (20.00–22.50)	< 0.001	0.518
Laboratory findings							
EOS (/uL)	130.00 (60.00–430.00)	100.00 (50.00–170.00)	0.469	250.00 (140.00–430.00)	130.00 (70.00–260.00)	0.003	0.087
EOS group (/uL)			1.000			0.001	0.041
<150	7 (63.64)	8 (72.73)		17 (26.98)	35 (55.64)		
≥150	4 (36.36)	3 (27.27)		46 (73.02)	28 (44.44)		
Serum total IgE (UI/mL)	608.00 (270.50–718.50)	642.00 (362.00–1071.00)	0.677	431.00 (232.50–1053.45)	664.00 (263.00–2380.00)	0.001	0.307
FeNO (ppb)	24.00 (22.00–47.00)	18.00 (16.00–23.00)	0.029	33.00 (20.00–61.00)	15.00 (8.00–26.00)	< 0.001	0.177
Lung function test							
Pre-BD FEV_1_ (%pred)	42.95 ± 16.65	48.77 ± 11.54	0.017	70.42 ± 22.01	77.85 ± 19.18	< 0.001	< 0.001
Post-BD FEV_1_ (%pred)	47.96 ± 17.17	54.01 ± 12.20	0.037	76.65 ± 22.52	84.87 ± 20.62	< 0.001	< 0.001
Post-BD FEV_1_/FVC (%)	61.10 (53.80–70.05)	62.60 (58.25–70.65)	0.833	93.20 (84.30–103.40)	99.45 (87.72–107.47)	0.003	< 0.001
Reversibility (%)	9.26 (2.52–28.51)	16.37 (7.03–18.18)	1.000	9.32 (3.32–14.82)	7.74 (4.90–12.19)	0.575	0.195

The data is presented in the form of mean ± standard deviation (normal distribution) and median (1st Quartile–3rd Quartile) (non normal distribution). *P* values of characteristics before and after omalizumab treatment were derived from the paired *t* test (the difference conforms to a normal distribution), the Wilcoxon signed rank test (the difference conforms to a non-normal distribution) and chi-square test or fisher exact test (for categorical variables). *P* values between two subgroups were derived from the Student’s t test (for normally distributed variables), Mann–Whitney test (for non-normally distributed variables) and chi-square test or fisher exact test (for categorical variables).

^#^For Fisher exact test.

ACO, asthma-chronic obstructive pulmonary disease overlap; ICS, inhaled corticosteroid; LABA, long-acting beta-agonist; IVGC, intravenous glucocorticoid; ACT, asthma control test; EOS, eosinophil; FeNO, fractional exhaled nitric oxide; Pre-BD FEV_1_, pre-bronchodilator forced expiratory volume in 1 s %; Post-BD FEV_1_, post-bronchodilator forced expiratory volume in 1 s %; Post-BD FEV_1_/FVC, post-bronchodilator forced expiratory volume in 1 s/forced vital capacity ratio.

**Figure 2:**
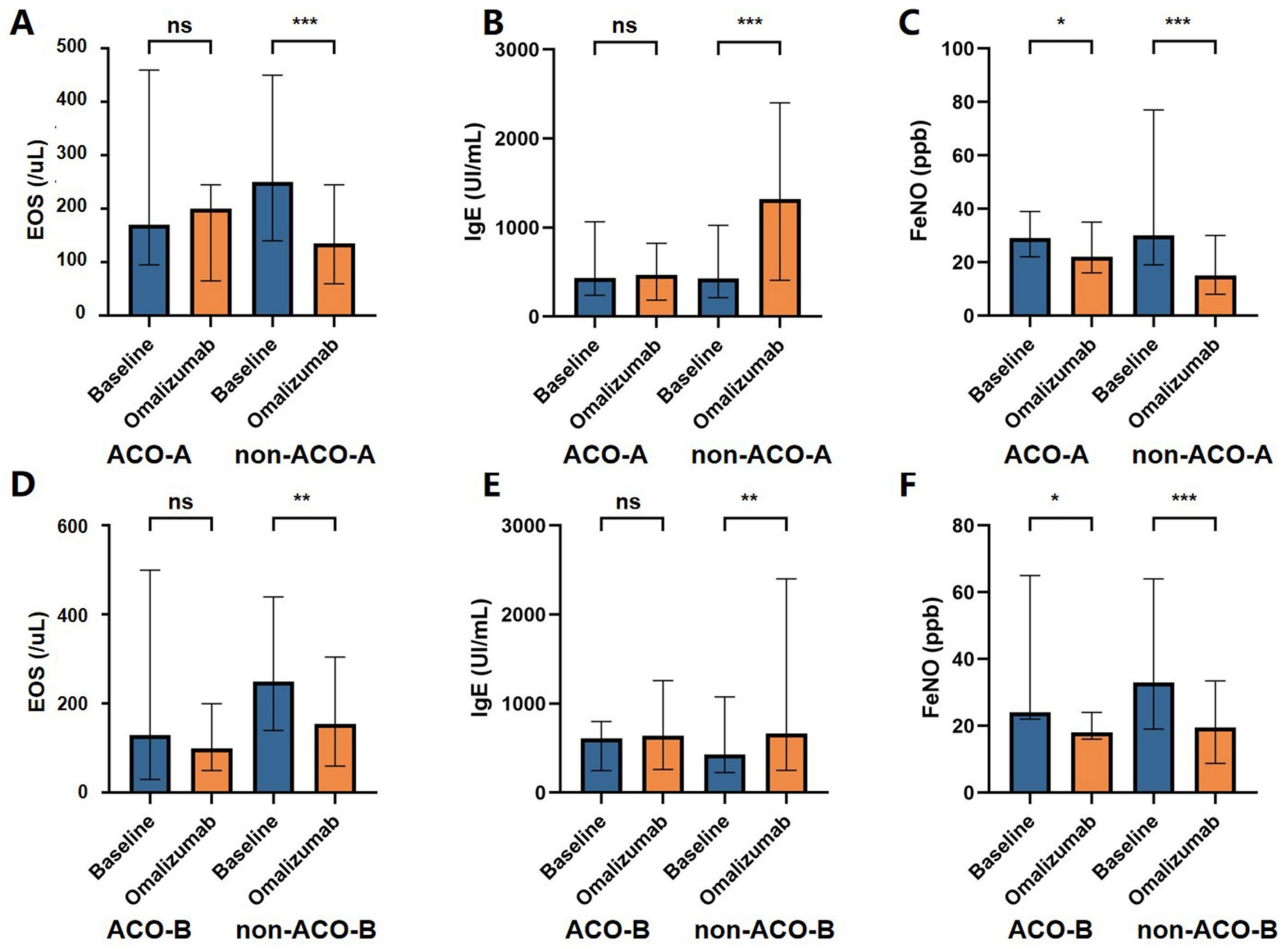
Changes in laboratory test indicators and FeNO of ACO and non-ACO asthma patients before and after 20 weeks treatment with omalizumab.

**Figure 3:**
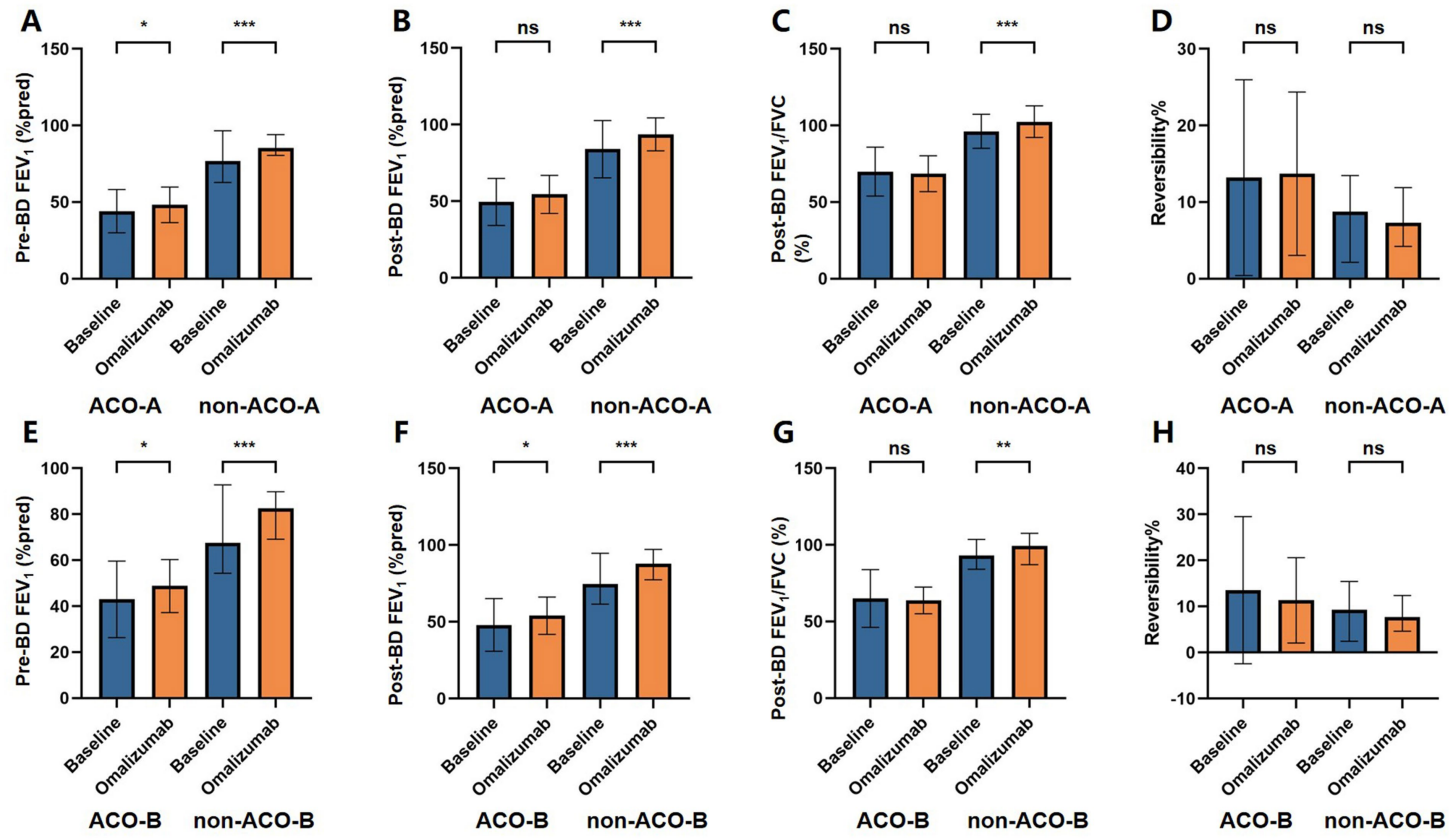
Changes in lung function test indicators of ACO and non-ACO asthma patients before and after 20 weeks treatment with omalizumab.

Following 20 weeks of omalizumab treatment, the proportion of individuals with EOS counts of ≥150 cells/μL showed a significant decrease from baseline in both the non-ACO-A (*p* < 0.001) and non-ACO-B (*p* = 0.001) groups. Both the ACO-A and ACO-B groups showed a significant decrease in FeNO levels [ACO-A: 29.00 (22.00–36.00) to 22.00 (13.50–29.00), *p* = 0.044; ACO-B: 24.00 (22.00–47.00) to 18.00 (16.00–23.00), *p* = 0.029]. Concurrently, pre-BD FEV_1_%predicted improved in both groups (ACO-A: 44.04 ± 14.14 to 47.47 ± 11.95, *p* = 0.035; ACO-B: 42.95 ± 16.65 to 48.77 ± 11.54, *p* = 0.017), and the ACO-B group also showed an increase in post-BD FEV_1_%predicted (47.96 ± 17.17 to 54.01 ± 12.20, *p* = 0.037). Serum total IgE [1320.00 (494.00–2380.00) vs. 431.00 (227.00–1020.00), *p* < 0.001], pre-BD FEV_1_%predicted (85.89 ± 9.49 vs. 77.71 ± 18.43, *p <* 0.001), post-BD FEV_1_%predicted (93.72 ± 10.76 vs. 84.03 ± 18.68, *p <* 0.001), and post-BD FEV_1_/FVC [100.10 (96.80–108.60) vs. 98.60 (86.90–103.70), *p* < 0.001] were significantly elevated relative to baseline in the non-ACO-A group, while FeNO significantly decreased [14.00 (8.00–22.00) vs. 30.00 (19.00–73.00), *p* < 0.001]. In the non-ACO-B group, patients also showed significantly lower FeNO [15.00 (8.00–26.00) vs. 33.00 (20.00–61.00), *p* < 0.001], higher serum total IgE [664.00 (263.00–2380.00) vs. 431.00 (232.50–1053.45), *p* = 0.001], pre-BD FEV_1_%predicted (77.85 ± 19.18 vs. 70.42 ± 22.01, *p* < 0.001), post-BD FEV_1_%predicted (84.87 ± 20.62 vs. 76.65 ± 22.52, *p* < 0.001) and post-BD FEV_1_/FVC [99.45 (87.72–107.47) vs. 93.20 (84.30–103.40), *p =* 0.003] compared to baseline. Figures 2, 3 illustrate the changes in these indicators before and after omalizumab treatment in the ACO-A, non-ACO-A, ACO-B, and non-ACO-B groups.

We further examined changes in EOS count, serum total IgE, FeNO level, pre-BD FEV_1_%predicted, post-BD FEV_1_%predicted, post-BD FEV_1_/FVC and reversibility from baseline to 20 weeks post-treatment in each subgroup. Details are presented in Supplementary Table 1. Notably, changes in serum total IgE and FeNO were more pronounced in the non-ACO-A group than in the ACO-A group (all *p <* 0.05). To assess the relationship between Th2 inflammatory levels and treatment outcomes, subgroup analyses were conducted and the details were showed in Supplementary Table 2. The subgroup with baseline EOS counts ≥ 150 cells/μL demonstrated a more pronounced reduction in EOS (*p* < 0.001) and FeNO (*p =* 0.012), alongside a greater increase in IgE (*p =* 0.003).

### 3.3 Comparison of post-treatment outcomes between ACO and non-ACO asthma patients

Comparative analysis of the corresponding subgroups after 20 weeks of omalizumab treatment is also summarized in Tables 2, 3. The non-ACO-A group demonstrated significantly higher pre-BD FEV_1_%predicted, post-BD FEV_1_%predicted and post-BD FEV_1_/FVC compared to the ACO-A group (all *p* < 0.001). A similar trend was observed between the non-ACO-B and ACO-B groups (all *p* < 0.001). Given the baseline age disparity between the ACO-A versus non-ACO-A groups, an ANCOVA adjusting for age was conducted to assess differences in outcomes between them. The adjusted results, presented in Table 4, confirmed that pre-BD FEV_1_%predicted, post-BD FEV_1_%predicted and post-BD FEV_1_/FVC remained significantly higher in the non-ACO-A group than in the ACO-A group after treatment (all *p* < 0.001).

**Table 4:** Comparison of post-treatment outcomes between ACO and non-ACO asthma patients.

Variables	Omalizumab			
	ACO-A (*n* = 25)	non-ACO-A (*n* = 49)	*F*	*P*
ACT scores	21.33 ± 0.49	21.22 ± 0.34	0.029	0.965
Laboratory findings				
EOS (/uL)	250.00 ± 50.00	180.00 ± 30.00	1.637	0.205
Serum total IgE (UI/mL)	707.84 ± 261.96	1530.69 ± 195.51	5.997	0.018
FeNO (ppb)	32.10 ± 5.28	20.51 ± 4.13	2.795	0.101
Lung function test				
Pre-BD FEV_1_ (%pred)	49.49 ± 2.27	84.69 ± 1.72	145.213	< 0.001
Post-BD FEV_1_ (%pred)	55.50 ± 2.47	92.16 ± 1.94	127.450	< 0.001
Post-BD FEV_1_/FVC (%)	67.71 ± 2.68	102.46 ± 2.10	97.291	< 0.001
Reversibility (%)	13.49 ± 2.03	8.65 ± 1.59	3.313	0.075

The data is presented in the form of adjusted mean ± standard deviation. *P* values between two subgroups were derived from the ANCOVA test (adjusted for age).

ACO, asthma-chronic obstructive pulmonary disease overlap; ACT, asthma control test; EOS, eosinophil; FeNO, fractional exhaled nitric oxide; Pre-BD FEV_1_, pre-bronchodilator forced expiratory volume in 1 s %; Post-BD FEV_1_, post-bronchodilator forced expiratory volume in 1 s %; Post-BD FEV_1_/FVC, post-bronchodilator forced expiratory volume in 1 s/forced vital capacity ratio.

## 4 Discussion

In this retrospective cohort study, we primarily aimed to compare treatment responses to omalizumab over 20 weeks between asthma patients with and without ACO. To account for diagnostic heterogeneity, our study incorporated two distinct ACO criteria, with the analysis of their impact constituting an exploratory aim. The differential treatment responses observed between ACO and non-ACO patients highlight the clinical challenge of applying asthma-related biological therapies to ACO, particularly when Th2-driven inflammation coexists with non-Th2 inflammatory pathways.

The clinical profile of ACO patients provides a tangible reflection of this pathophysiological complexity. The baseline characteristics of ACO patients, characterized by poorer lung function and a higher proportion of smokers compared to those with non-ACO asthma, provide a crucial context for interpreting the differential responses to omalizumab observed in our study. These pre-existing differences suggest that the distinct pathophysiology of ACO may underlie its modified response to Th2-targeted therapy.

As an anti-IgE monoclonal antibody, omalizumab inhibits IgE from binding to its receptor on mast cells and basophils, thereby preventing their activation, reducing the release of cytokines such as IL-5, and suppressing eosinophil recruitment and activation (25). This mechanism underpins its targeted anti-inflammatory effect in Th2-high driven disease. A recent long-term retrospective study defined reductions in FeNO and EOS counts as integral components of a “complete response” to biologics in severe asthma (20). Our observations are in alignment with this definition, demonstrating a significant decrease in both biomarkers following omalizumab treatment, which reflects the core biological mechanism of omalizumab in targeting Th2 inflammation (26). Furthermore, our subgroup analysis based on baseline peripheral blood EOS counts provided deeper insights, revealing that the magnitude of eosinophilic inflammation was a pivotal determinant of the treatment response to anti-IgE therapy. Specifically, patients with baseline blood EOS counts ≥150 cells/μL demonstrated a significantly stronger treatment response to omalizumab compared to those with values below this threshold. This suggests that assessing eosinophilic inflammation may hold greater predictive value for omalizumab responsiveness than the broad syndromic classification of ACO alone, underscoring a personalized, biomarker-driven approach. Consequently, our findings add to the body of evidence supporting the role of EOS and FeNO as potential biomarkers for identifying patients likely to benefit from omalizumab treatment (27–32). However, the predictive value of FeNO and EOS as standalone biomarkers remains limited (25, 33). Therefore, identifying more robust biomarkers to predict omalizumab efficacy remains an ongoing priority. This endeavor has expanded to include candidates such as serum periostin, a downstream marker of IL-13 activity (34), dynamic changes in total IgE levels early in treatment (35), and markers of eosinophilic inflammation in the upper airways (36).

In addition to exploring new candidates, our findings call for a re-evaluation of classic biomarkers, particularly total IgE, in the context of treatment. Our study observed a significant increase in serum total IgE levels in non-ACO asthma patients after omalizumab initiation, a finding that contrasts with certain previous reports (37). This apparent discrepancy can be explained by its mechanism: omalizumab binds to free IgE, forming large, stable complexes that are detected in standard assays, leading to a transient elevation in measured total IgE during early treatment—even as bioactive free IgE declines substantially (30, 37, 38). Thus, the observed rise in total IgE does not indicate treatment failure but rather reflects expected pharmacological activity. Consequently, serum total IgE proves to be a suboptimal short-term efficacy biomarker for omalizumab (39, 40).

Beyond the monitoring of biomarkers, the evaluation of therapeutic response should prioritize tangible clinical improvements, such as symptom control and reduced exacerbation frequency. In our study, both ACO and non-ACO groups showed improved symptom control and reduced exacerbations. However, longitudinal improvement in biomarkers and lung function was observed only in the non-ACO group, a finding consistent with the Australian Xolair Registry report that omalizumab improved quality of life but not lung function in ACO patients (41). This underscores that for ACO patients, the primary treatment goal may not be to reverse lung function decline but to maintain disease stability and reduce the burden of life, an objective supported by a decrease in exacerbation rates and hormone use. This is corroborated by a *post hoc* analysis of the PROSPERO trial, which found both groups experienced improvements in ACT scores and exacerbation rates (42). Therefore, although omalizumab demonstrates lower efficacy in ACO than in non-ACO patients, it remains a viable therapeutic option for symptom management in this population (43, 44).

The observed heterogeneity in treatment response underscores the importance of how ACO is defined. Our exploratory analysis using two distinct diagnostic criteria provided further insight. The significant differences in longitudinal changes of FeNO, IgE and pre-BD FEV_1_%pred between groups under criterion A, but not under criterion B, suggest that these criteria may select for patient populations with differing underlying inflammatory endotypes. Criterion A, with its emphasis on clinical history, might better identify an “asthma-dominant” ACO phenotype, characterized by a stronger Th2 component, which is consequently more responsive to Th2-targeted therapy like omalizumab. In contrast, criterion B, which incorporates fixed airflow limitation and smoking history, might capture a “COPD-dominant” ACO phenotype where non-Th2 inflammatory pathways such as neutrophilic inflammation play a more dominant role, explaining the attenuated response. This supports the concept of ACO as an umbrella term for mixed inflammation (45), encompassing subtypes like smoking asthmatics (SA) and COPD with eosinophilia (COPD-e) (46). Consequently, the Th2 inflammatory endotype and its associated biomarkers (e.g., EOS, FeNO) are more informative predictors of omalizumab responsiveness than the broad syndromic classification of ACO itself (47). Therefore, in clinical practice, evaluating a patient’s individual inflammatory burden may hold greater value for biologic selection than the specific ACO definition used.

This study provides a multidimensional comparison of omalizumab efficacy in ACO versus non-ACO asthma, stratified by two diagnostic criteria. However, several limitations should be considered. Firstly, the retrospective design, limited sample size, and short follow-up duration constrain the generalizability and assessment of long-term outcomes. Secondly, the use of the fixed-ratio (FEV_1_/FVC < 0.70) for ACO-B classification, while clinically pragmatic, may less accurately define airflow limitation compared to the lower limit of normal (LLN). Finally, the absence of functional validation (e.g., bronchoscopic biopsies) limits causal inferences from biomarker correlations. Consequently, future research requires larger longitudinal cohorts to validate diagnostic criteria and confirm response durability, incorporate free IgE measurements to clarify the significance of post-treatment total IgE elevation, and employ direct tissue sampling to mechanistically link Th2 inflammation reduction to clinical improvement.

## 5 Conclusion

Our study clearly indicates that the presence of a Th2-high inflammatory endotype—not the specific ACO diagnostic label—is the key predictor of omalizumab response. While all patient groups experienced clinical benefits from omalizumab, including reduced exacerbations and improved symptom control, the degree of improvement in biomarkers and lung function was most pronounced in non-ACO asthma patients and those identified by the COPD history-based ACO criterion, which effectively selects for a stronger Th2 phenotype. Prioritizing direct assessment of Th2 inflammation, rather than relying on the ACO definition, provides a better guide for selecting biologic therapy.

## Data Availability

The original contributions presented in the study are included in the article/Supplementary material, further inquiries can be directed to the corresponding authors.
