# Supplementary material for: Comparative effectiveness of omalizumab in asthma-COPD overlap vs. asthma: a retrospective cohort study
Source: Front Med (Lausanne). 2026 Jan 21;13:1738610. doi: 10.3389/fmed.2026.1738610 (PMC12868278; doi:10.3389/fmed.2026.1738610)
Supplement: Supplementary file 1 [file Data_Sheet_1.docx]

**Comparative Effectiveness of Omalizumab in Asthma- COPD Overlap vs. Asthma: A Retrospective Cohort Study**

**Supplementary Tables**

**Supplementary Table 1** Comparison of characteristic changes in ACO and asthma patients before and after treatment with Omalizumab.

| **Variables** | **Total (n = 74)** | **ACO-A**  **(n = 25)** | **non-ACO-A**  **(n = 49)** | ***P*** | **ACO-B**  **(n = 11)** | **non-ACO-B**  **(n = 63)** | ***P*** |
| --- | --- | --- | --- | --- | --- | --- | --- |
| ΔEOS (/uL) | -90.00 (-250.00–0.00) | -30.00 (-30.00–10.00) | -120.00 (-250.00–0.00) | 0.140 | -20.00 (-300.00–10.00) | -90.00 (-240.00–0.00) | 0.523 |
| ΔIgE (UI/mL) | 141.40 (5.10–756.50) | 69.50 (-14.83–182.40) | 432.66 (6.80–1210.00) | 0.011 | 48.00 (-64.00–182.40) | 298.00 (6.80–972.00) | 0.078 |
| ΔFeNO (ppb) | -11.00 (-33.00–-6.25) | -7.00 (-22.50–-0.50) | -16.00 (-37.00–-9.00) | 0.008 | -7.00 (-17.50–-5.00) | -12.00 (-34.50–-7.50) | 0.203 |
| ΔPre-BD FEV_1_ (%pred) | 8.85 ± 12.61 | 4.59 ± 8.76 | 11.39 ± 13.94 | 0.062 | 5.82 ± 6.78 | 9.69 ± 13.74 | 0.203 |
| ΔPost-BD FEV_1_ (%pred) | 6.20 (2.30–15.80) | 3.00 (-0.05–13.65) | 9.15 (4.45–17.08) | 0.063 | 4.30 (0.70–13.65) | 7.45 (2.85–17.08) | 0.270 |
| ΔPost-BD FEV_1_/FVC (%) | 3.20 (0.00–7.20) | 2.10 (-7.45–8.40) | 3.45 (0.05–7.07) | 0.241 | 3.20 (0.65–8.40) | 3.20 (0.00–7.07) | 0.848 |
| ΔReversibility (%) | -9.58 (-16.75–-2.14) | -9.08 (-25.02–-2.55) | -9.60 (-15.02–-2.81) | 0.722 | -9.08 (-28.44–-2.55) | -9.60 (-15.31–-2.81) | 0.768 |

**Abbreviations**: ACO: Asthma-chronic obstructive pulmonary disease overlap; BMI: Body mass index; EOS: Eosinophil; FeNO: Fractional exhaled nitric oxide; Pre-BD FEV_1_: Pre-bronchodilator forced expiratory volume in 1 second %; Post-BD FEV_1_: Post-bronchodilator forced expiratory volume in 1 second %; Post-BD FEV_1_/FVC: Post-bronchodilator forced expiratory volume in 1 second / forced vital capacity ratio.

**Supplementary Table 2** Changes in clinical parameters following omalizumab treatment stratified by baseline EOS levels.

| **Variables** | **EOS subgroup** | | |
| --- | --- | --- | --- |
|  | **<150/uL** | **≥150/uL** | ***P*** |
| Δ ***ACT scores*** | 3.00 (1.00–4.00) | 2.50 (1.00–5.75) | 0.780 |
| ***Laboratory findings*** |  |  |  |
| Δ EOS (/uL) | 10.00 (-20.00–40.00) | -180.00 (-300.00–-80.00) | <0.001 |
| Δ Serum total IgE (UI/mL) | 69.50 (0.00–141.40) | 373.00 (55.00–1210.00) | 0.003 |
| Δ ***FeNO (ppb)*** | -8.00 (-13.75–-2.75) | -17.50 (-46.25–-8.00) | 0.012 |
| ***Lung function test*** |  |  |  |
| Δ Pre-BD FEV_1_ (%pred) | 13.74 ± 11.57 | 6.19 ± 12.52 | 0.040 |
| Δ Post-BD FEV_1_ (%pred) | 13.00 (7.15–16.78) | 4.30 (1.85–12.65) | 0.050 |
| Δ Post-BD FEV_1_/FVC (%) | 4.85 (2.30–11.08) | 2.10 (0.00–5.20) | 0.084 |
| Δ Reversibility (%) | -4.26 (-10.27–2.78) | 0.68 (-4.27–5.41) | 0.190 |

**Abbreviation**: ACO: Asthma-chronic obstructive pulmonary disease overlap; ACT: Asthma control test; EOS: Eosinophil; EOS: Eosinophil; FeNO: Fractional exhaled nitric oxide; Pre-BD FEV_1_: Pre-bronchodilator forced expiratory volume in 1 second %; Post-BD FEV_1_: Post-bronchodilator forced expiratory volume in 1 second %; Post-BD FEV_1_/FVC: Post-bronchodilator forced expiratory volume in 1 second / forced vital capacity ratio.
